# Clinical and Economic Impact of a Multidisciplinary Follow-Up Program in Lymphoma Patients

**DOI:** 10.3390/cancers14102532

**Published:** 2022-05-21

**Authors:** Madeline Devaux, Mathieu Boulin, Morgane Mounier, Denis Caillot, Nuri Ahwij, Adélie Herbin, Jean Noël Bastie, Camille Favennec, Philippine Robert, Pauline Pistre, Stephanie Bost, Pauline Amiot, Laurence Jacquesson, Olivier Casasnovas, Cédric Rossi, Pauline Gueneau

**Affiliations:** 1 Department of Pharmacy, University Hospital, F-21000 Dijon, France; pauline.pistre@chu-dijon.fr (P.P.); stephanie.bost@chu-dijon.fr (S.B.); pauline.gueneau@chu-dijon.fr (P.G.); 2 Department of Pharmacy, University Hospital and EPICAD LNC UMR1231, University of Burgundy & Franche Comte, F-21000 Dijon, France; mathieu.boulin@chu-dijon.fr; 3 Registre des Hémopathies Malignes de Côte d’Or, Dijon-Bourgogne University Hospital, F-21000 Dijon, France; morgane.mounier@chu-dijon.fr; 4 INSERM, U1231, University of Burgundy & Franche Comte, UMR 1231, F-21000 Dijon, France; 5 Department of Clinical Hematology, University Hospital INSERM UMR1231 and SAPHIIR-UMR 1231, University of Burgundy & Franche Comte, F-21000 Dijon, France; denis.caillot@chu-dijon.fr (D.C.); olivier.casasnovas@chu-dijon.fr (O.C.); 6 Department of Clinical Hematology, University Hospital, F-21000 Dijon, France; nuri.ahwij@chu-dijon.fr (N.A.); adelie.herbin@chu-dijon.fr (A.H.); jean-noel.bastie@chu-dijon.fr (J.N.B.); camille.favennec@chu-dijon.fr (C.F.); philippine.robert@chu-dijon.fr (P.R.); pauline.amiot@chu-dijon.fr (P.A.); laurence.jacquesson@chu-dijon.fr (L.J.); 7 Department of Clinical Hematology, University Hospital and SAPHIIR-UMR 1231, University of Burgundy & Franche Comte, F-21000 Dijon, France; cedric.rossi@chu-dijon.fr

**Keywords:** lymphoma, follow-up, nurse, pharmacist, immunochemotherapy, haematotoxicity, quality of life

## Abstract

**Simple Summary:**

The treatment of Hodgkin and non-Hodgkin lymphoma is mainly based on highly haematotoxic chemoimmunotherapy regimens that can cause serious adverse events (AEs). We hypothesised that scheduled phone calls by a nurse combined with the intervention of the pharmaceutical team can reduce the frequency of AEs and their consequences. Thus, the UMACOACH Lymphoma Program (ULP) was created in 2019 in our institution. The primary objective of our study was to evaluate the clinical and economic impact of the ULP compared to a matched cohort of patients managed before the start of ULP. The secondary objective was to assess patient satisfaction and quality of life (QoL). Our study highlights the positive impact of a triple nurse–pharmacist–hematologist collaboration in reducing AEs and re-hospitalisations through earlier detection of symptoms and better management of patients’ supportive care at home, as well as patient satisfaction and improved quality of life.

**Abstract:**

Objectives: The UMACOACH Lymphoma is a multidisciplinary monitoring program for patients initiating a first highly haematotoxic treatment for Hodgkin or non-Hodgkin lymphoma. Patient follow-up is based on consultation with a pharmacist and planed phone calls by nurses supervised by a clinical haematologist. Our objective was to assess effectiveness and cost of the UMACOACH Lymphoma Program (ULP) and to investigate patient satisfaction and quality of life (QoL). Methods: This French monocentric case-control study included all patients enrolled in the ULP over a one-year period (cases) matched with retrospective patients receiving usual care (controls). Numbers of adverse events (AEs), re-hospitalisations, average relative dose intensity (ARDI), treatment response and survival were compared between the two groups. Among cases, patient satisfaction and QoL using the EORTC-QLQC30 questionnaire before and after treatment were evaluated. Results: Seventy-eight cases were matched to 78 controls. Twenty-six percent grade 3–4 AEs were observed in cases versus 38% in controls (*p* = 0.001). There were 76 and 88 re-hospitalisations in the case and control groups, respectively (*p* = 0.217). ARDI > 85% was observed in 92% and 82% of cases and controls, respectively (*p* = 0.138). No differences were observed in terms of treatment responses and survival. Estimated cost savings were of EUR 81,782 in favour of the case group. An improvement of 5.1 points was observed in the total QoL score before and after treatment in cases. Conclusions: A nurse–pharmacist–haematologist collaboration seems to be promising to reduce grade 3–4 AEs in HL and NHL patients receiving highly haematotoxic chemotherapy regimens. Cost savings from hospitalisation being avoided were also shown.

## 1. Introduction

**Figure 1:**
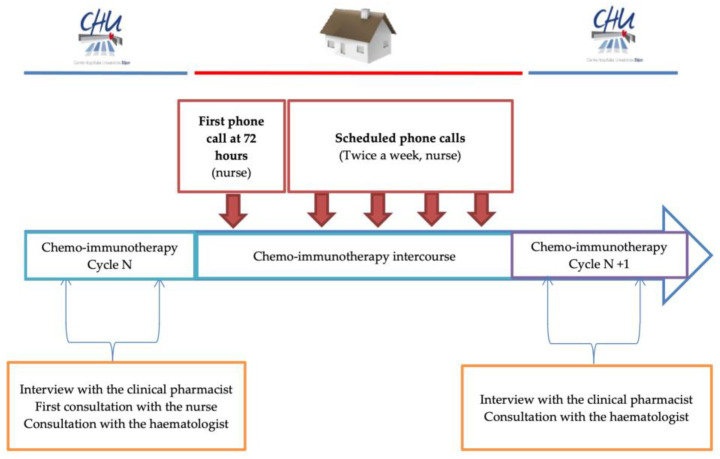
Diagram of the care pathway of the patient participating in the UMACOACH Lymphoma Program.

Hodgkin lymphoma (HL) and non-Hodgkin lymphoma (NHL) treatment is mostly based on chemo-immunotherapy regimens. In Western countries, the most common subtype of NHL is diffuse large B cell lymphoma (DLBCL) [1]. R-CHOP (rituximab, cyclophosphamide, doxorubicin, vincristine, prednisone) is the standard-of-care for DLBCL first-line treatment based on the LNH-98-5 [2], and it is considered the backbone of therapy for other NHL sub-types such as follicular lymphoma (FL) and mantle cell lymphoma (MCL) followed by maintenance therapy with rituximab [3,4]. Some variants of R-CHOP, such as R-ACVBP (rituximab, doxorubicin, cyclophosphamide, vindesine, bleomycin, prednisone) or R-miniCHOP have been described for young in the GELA study [5,6] and older patients, respectively [7,8]. For HL, the standard of care in France is a chemotherapy regimen, namely, ABVD (doxorubicin, bleomycin, vinblastin, dacarbazine) [9] or escalated BEACOPP (cyclophosphamide, doxorubicin, etoposide, procarbazine, prednisone, vincristine and bleomycin) [3,10,11] according the prognostic factors and staging at diagnosis. All strategies were guided by Position Emission Tomography-Computed Tomography (PET-CT) results [12]. All these highly haematotoxic chemo-immunotherapy regimens lead to potentially serious adverse effects (AEs) related to additional costs and decreased treatment relative dose intensity (RDI) [13,14]. Serious AEs are usually managed by patients’ phone calls. However, these unscheduled phone calls may not resolve all AEs as they are not anticipated (patients advanced in their symptoms and/or biological abnormalities); they lack reliability involving many different healthcare professionals; they also disturb the routine hospital organisation. We hypothesised that scheduled phone calls performed by a nurse may decrease the frequency of serious haematologic AEs and its consequences (including hospitalisations) in anticipating their onset. To improve and standardise outpatient care, the UMACOACH Lymphoma Program (ULP) was developed in 2019 in our institution, which is one of the expert centres for lymphoma treatment in France (Figure 1). Pharmacists and nurses play a key role in this program, working closely with the medical team. During the first chemo-immunotherapy treatment, pharmacists have an individual meeting with the patient to explain treatment and related AEs. They also perform a full medication review to detect inappropriate medicines [15]. In addition to the usual medical management and monitoring, patients receive pre-planned calls from the ULP-dedicated nurse. 

The primary objective of our study is to assess the clinical and economic impact of the ULP compared to a matched cohort of patients managed before ULP onset. The secondary objective was to evaluate patient satisfaction and quality of life (QoL).

## 2. Materials and Methods

### 2.1. Study Design and Population 

Our study was monocentric, case-control in which all consecutive patients were included from 1 May 2019 to 30 April 2020 in the ambulatory haematology-oncology department of the Dijon University Hospital. Eligible patients were older than 18 years, with histologically confirmed non-Hodgkin or classical HL according to the WHO classification [16]. Patient exclusion criteria were inability to answer or no access to telephone, psychiatric illness or dementia. All patients provided written informed consent and the study procedures were in accordance with the revised Declaration of Helsinki (2008). In order to collect all relevant information, only patients who had completed the entire active treatment phase by 30 April 2020 were studied. Patients’ sociodemographic, disease and treatment data were recorded from the beginning of chemotherapy. 

### 2.2. Description of the UMACOACH Lymphoma Program

**Figure 2:**
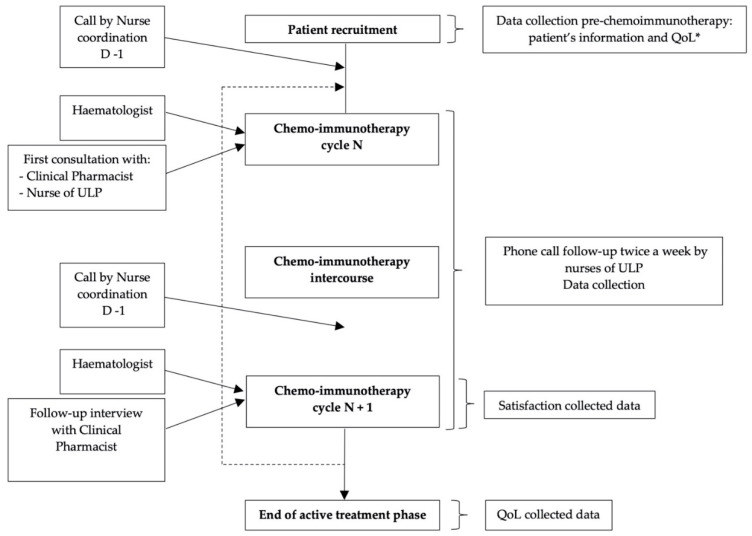
Description of UMACOACH Lymphoma Program. * QoL: Quality of Life; ULP: UMACOACH Lymphoma Program.

The description of the ULP is detailed in Figure 2.

#### 2.2.1. Nurse Intervention

Two half-time nurses were recruited for the ULP based on their motivations and their experiences in haematology (>20 years for the first one, 5 years for the second one). During the first course of chemo-immunotherapy, the nurses met the patient and described the ULP modalities and the follow-up procedures (hospitalisation days, blood test modalities and days, detection, prevention of AEs, and the emergency call procedure). Between two courses, each patient received two phone calls per week. Each call lasted about ten minutes. It consisted in a series of systematic questions. The phone follow-up started for all patients the third day after the first chemo-immunotherapy administration (first phone call) and stopped at the end of the active treatment phase or at the relapse time. During phone calls, the nurses filled out a standardised questionnaire with biological analyses, and AEs were graded according to the NCI CTCAE criteria and all other relevant data [17]. Nurses were trained to detect, rank and manage AEs according to a table and a decision tree validated by two haematologists. Nurses’ interventions were defined in 3 different grades: grade 0 in case of no intervention; grade 1 if an intervention was managed by the nurses through the decision tree; and grade 2 if the intervention required a haematologist intervention (symptoms requiring further investigation or in case of life-threatening complications). 

#### 2.2.2. Pharmacist Interventions (PI)

During patient interviews or medication review, clinical pharmacists performed pharmacist interventions (PIs), defined as “any action taken by a pharmacist that directly results in a change of patient management or therapy” [18], and they did so according to the following two modalities: PIs with prescribers (PIpr) and PIs with the patient during an interview (PIpa), as classified according to the validated tool from the French Society of Clinical Pharmacy (FSCP) [19]. The economic and clinical impact of PIs was assessed using the Clinical Economic and Organizational (CLEO) tool [20,21]. Each PI was first rated prospectively by the pharmacist who performed the PI. A second independent rating by two clinical pharmacists specialised in haematology and oncology was then performed. The third clinical pharmacist who rated the PI had 12 years of experience in haematology and oncology. 

### 2.3. Clinical and Economic Evaluation 

To analyze clinical and economic impact of the ULP, we matched patients prospectively included in the ULP with retrospective controls treated in our center before ULP onset and selected over the period from January 2015 to April 2019. Controls were identified from the population-based registry for haematological malignancies in the Côte d’Or area and from the hospital data of our clinical haematology research department. One for one matching was performed using a propensity score, coupled on individual and disease characteristics with an accurate matching on chemo-immunotherapy regimens. 

#### 2.3.1. Clinical Impact 

The clinical impact of the ULP was evaluated according to all grades and grade ≥ 3 AEs, re-hospitalisations between two courses, RDI, treatment response, progression free survival (PFS) and overall survival (OS). There were no modifications in prescribing practices over the two periods (for cases and controls) for supportive care agents, anti-emetics, antibiotic prophylaxis, Granulocyte-Colony Stimulating Factors (G-CSF) (administered if PNN count is ≤1 G/L and after decision of the haematologist based on the recommendations of the EORTC and ASCO [22,23]) and erythropoiesis-stimulating agents (ESA) (started if hemoglobin count ≤ 10 g/dL several times). 

RDI, calculated as previously described in Hryniuk et al. in 1984 for cyclophosphamide and doxorubicin [24], represents the dose of one specific drug administered over the total chemotherapy period, divided by the standard dose intensity specified in the protocol. Next, the averaged RDI (ARDI) was calculated by averaging the RDI of cyclophosphamide and/or doxorubicin in all the chemo-immunotherapy courses [25]. In our study, we considered the RDI to be satisfactory above 85% based on the study of Lyman et al. [26]. A delay higher than 7 days between two chemotherapy courses was also collected.

Treatment response criteria was evaluated by PET-CT during and at the end of the active treatment phase and defined according to the revised recommendations established by the International Working Group response criteria in 2017 [27]. PFS was defined as the time between the 1st chemotherapy day and the date of the 1st progression, relapse or death from any cause or loss of follow-up. OS was defined as the time between the 1st chemotherapy day and death from any cause or loss of follow-up. The last collection date was extended to 30 April 2021. 

#### 2.3.2. Economic Impact

The economic impact of the ULP, calculated from the cost of hospitalisations for complications related to treatment or disease during the active treatment phase, was measured using the French public health insurance system. Each hospitalisation in our institution was identified using the French national hospital discharge abstract database (Programme de médicalisation des systèmes d’information; PMSI) [28], allowing one to determine an all-inclusive cost covered by the health insurance (Homogeneous Stay Group (GHS)) for the various hospitalisations. Cost of implementation of the ULP included costs of a full-time nurse (or two half-time nurses; EUR 50,000/year) and of a 0.2 full-time clinical pharmacist based on its activities dedicated to the program in the department (EUR 14,000/year).

### 2.4. Quality of Life and Patient Satisfaction

QoL was assessed using the EORTC QLQ C30 questionnaire established in 1986 by the European Research Organization of Cancer Treatment [29,30]. Each patient filled out the questionnaire independently before and after the active treatment phase. In order to demonstrate a significant clinical difference, the Minimal Clinically Important Difference (MCID) value was calculated and was considered significant beyond 10 points [31]. A patient standardised satisfaction questionnaire, consisting of 12 multiple-choice questions, was proposed to all patients included in the ULP who had received at least one course of chemo-immunotherapy. 

### 2.5. Statistical Analyses

Quantitative variables were described using medians and ranges and compared with Student or Mann–Whitney tests. Qualitative variables were described using frequency and percentages and compared with chi-squared or Fisher exact tests. The Wilcoxon test was used to compare statistical difference in the QoL change. The MCID value was compared to the theorical level defined by Osoba et al. in 1998 to estimate clinical impact [31]. OS and PFS were estimated until 18 months after lymphoma diagnosis using the Kaplan–Meier non-parametric estimator. Survival distributions were statistically compared using the Log-rank test. Analyses were performed using R software, version 3.6 (R Foundation for Statistical Computing, Vienna, Austria). Significant level was fixed at 0.05.

## 3. Results

**Figure 3:**
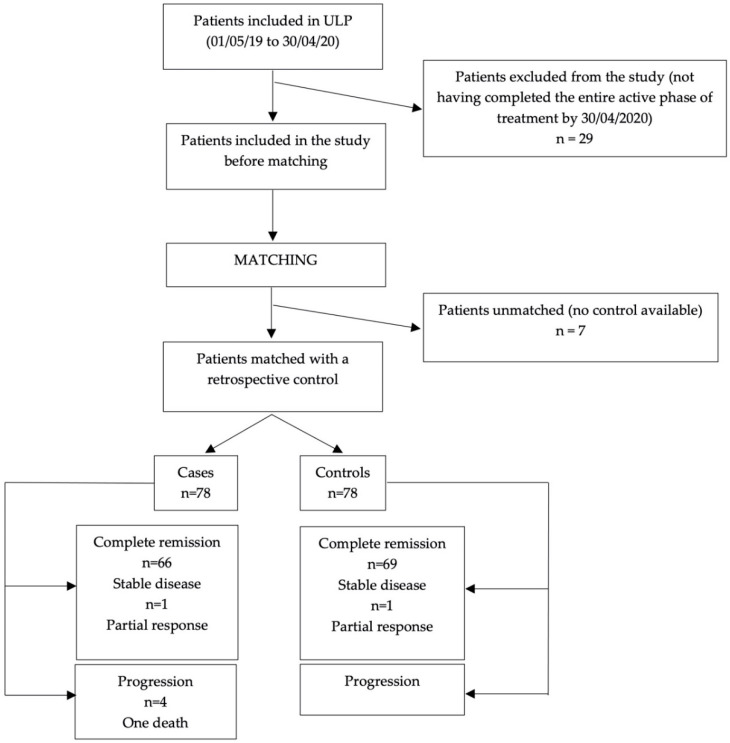
Flowchart.

One hundred and fourteen patients were included in the ULP over the one-year recruitment period. Patient baseline characteristics are detailed in Table 1. Among them, 78 were matched to controls (Figure 3, Table 2).

### 3.1. ULP Description 

Overall, 3075 phone calls with a median of 38 [11–65] calls/patient were performed, which accounted for a total of 512.5 nursing hours. Phone calls generated 2609 (85%) “grade 0” interventions, 420 (14%) “grade 1” interventions and 46 (1%) “grade 2” interventions. 

Three hundred PIs (115 (38%) PIpr and 185 (62%) PIpa) were performed, corresponding to a median of 3 [1–8] PIs/patient (Table 3). The most common “drug related problem” was “drug or medical device not received by the patient” noted in 36% which resulted in “addition of a new drug” (39%). The clinical impact of PIs was mostly minor for 219 (73%), but 16 (5%) PIs were classified as major, meaning that they had potentially avoided an iatrogenic hospitalisation. PIs were considered “major” when they directly affected the dose of the chemo-immunotherapy, for example, increasing uromitexan dose adapted to cyclophosphamide dose or adapting vincristine dose to the patient’s age.

### 3.2. Clinical Impact

Five hundred and sixty-nine and 513 chemo-immunotherapy were, respectively, performed in the case and the control groups, respectively. The percentage of patients receiving ESA was significantly higher in the case group (*p* = 0.001). Overall, 465 and 343 all-grade AEs were identified in the case and control groups, respectively. Twenty-six percent were grade 3–4 AEs in cases versus 38% in controls (*p* = 0.001). The number of grade 3–4 infections without neutropenia was significantly lower in cases (*p* = 0.038). Thirty-five cases and 37 controls required at least one re-hospitalisation between two courses of chemo-immunotherapy in relationship with anti-lymphoma treatment. Overall, 76 re-hospitalisations (29% in haematology department) and 88 re-hospitalisations (39% haematology department, 1% in an intensive care unit) were identified in the case and control groups, respectively (Table 4 and Table 5).

ARDI could not be calculated for 5 of 78 cases and for 5 of 78 controls because chemo-immunotherapy regimens did not include cyclophosphamide or doxorubicin. ARDI was below 85% for 6 (8%) patients versus 13 (18%) patients in the case and control groups, respectively (*p* = 0.138). A delay higher than 7 days between two chemotherapy courses was observed in 23 (32%) patients in the case group and in 24 (33%) patients in the control groups. 

Treatment response was assessed in the 78 matched case/control population. In the case and control groups there were, respectively, 66 (85%) and 69 (88%) patients to achieve a CR; 7 (9%) and 4 (5%) went into a PR and 1 (1%) and 1 (1%) had a stable disease. Four (5%) patients in the cases and the controls progressed “on therapy” requiring a relapse treatment. The ULP may not influence the treatment response since no statistically significant difference between the case and control group was observed (*p* = 0.515).

**Figure 4:**
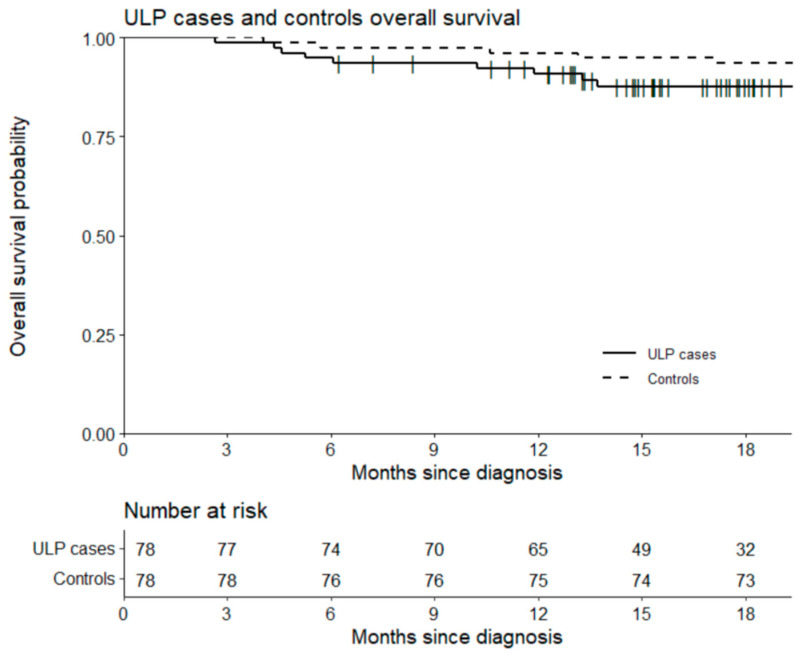
Overall survival of cases (including in ULP) and control groups.

**Figure 5:**
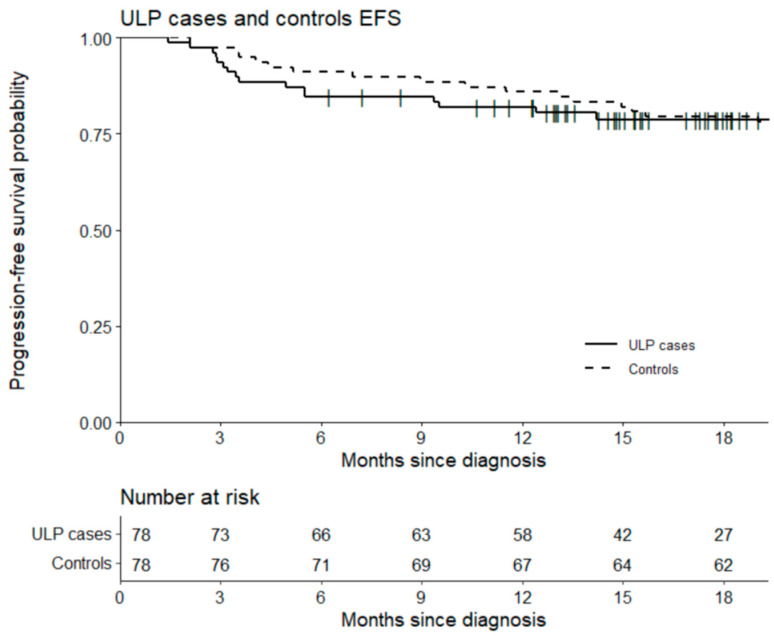
Progression-free survival of cases (including in ULP) and control groups.

The median patient follow-up was 18 months. The 18-month PFS was equal to 79.5% in the two study groups with 16 and 15 relapses in the case and the control groups, respectively (*p* = 1). Although not significant, 18-month OS was higher in the control group (94.9% vs. 88.5%) (*p* = 0.25). There were nine lymphoma-related deaths (11.5%), and four deaths (two related to lymphoma, one due to hypoxic cardiac arrest, one unknown cause) (5.1%) in the case and the control groups, respectively (Figure 4 and Figure 5).

### 3.3. Economic Impact

The total cost of re-hospitalisation was EUR 166,299 and EUR 248,081 for cases and controls, respectively. The median cost per patient was EUR 2807 [495–25,493] in the case group vs. EUR 4192 [586–33,089] in the control group (*p* = 0.564). Thus, there was a cost saving of EUR 81,000 for the French public health insurance in favour of the case group. The cost of the implementation of the ULP was EUR 64,000/year for a final positive margin of EUR 17,000/year. 

### 3.4. Quality of Life and Satisfaction

#### 3.4.1. Satisfaction

Eighty patients (70%) filled out the satisfaction questionnaire (Table 6). Ninety-nine percent declared feeling more confident and reassured by the phone calls. Listening, the time devoted to them as well as the quality of responses corresponded for the totality of patients as much as they expected. For 95% of patients, the interaction with the pharmacist was judged satisfactory or very satisfactory. With a better understanding of their treatment, patients would recommend the ULP program to other patients. 

#### 3.4.2. Quality of Life

Twenty-nine patients filled out the questionnaires before and after the active treatment phase. The distribution of scores was established for each item (Table 7). All items of functional scale increased, one with a clinical significativity of 12.1 points for the “role functioning” item (*p* = 0.077). Concerning the symptom scale, a decreased score was observed for all items, in particular for pain, fatigue, appetite loss and diarrhoea; only dyspnea score increased. An increase of 3.3 points was noted for the global health status. Finally, for all items an increase of 5.1 points in QoL was observed (*p* = 0.199). 

## 4. Discussion

A personalised multidisciplinary follow-up program dedicated to patients undergoing highly haematotoxic chemo-immunotherapy regimens for lymphoma is efficient compared to usual care (control group), with significantly fewer grade ≥ 3 infections and less re-hospitalisations, cost-savings, and patient satisfaction. 

One of the major benefits of the phone calls was the close relationship established between the nurses and the patients, allowing nurses to collect more information on their global health status, to anticipate deteriorations and the occurrence of serious grade ≥3 AEs. Phone calls were managed by experienced nurses helped by medically validated decision trees, freeing up the precious time of the haematologist, who was previously involved in responding to patients by phone.

Thanks to their interventions, nurses detected AEs earlier (26% of grade 3–4 toxicities in the case group versus 38% in the control group) and more exhaustively (465 in the cases versus 343 in the controls), therefore limiting the risk of developing serious AEs. An example to illustrate this benefit is the lower percentage of grade 3–4 anemia in the ULP group in relation with a higher percentage of patients receiving ASE (40%) (*p* = 0.001). Similarly, the percentage of grade 3–4 infections was significantly lower in the case group (6%) than in the control group (15%) (*p* = 0.038). Finally, the lower number of re-hospitalisations to receive intravenous antimicrobial agents or blood transfusions in the cases highlighted the positive ULP impact for patient health.

By limiting the occurrence of grade ≥3 AEs, the ULP probably contributes to optimised anti-lymphoma efficacy. However, there was not a significant difference between the ARDI > 85% in the cases and controls (ARDI < 85% for 8% of cases versus 18% of controls, *p* = 0.138). This result is better than that described in a study conducted in 2004 in the United States by Lyman et al., showing that 40% of patients treated with R-CHOP had an RDI < 85% [26]. However, this result was described before the routine implementation of G-CSF prophylaxis. According to another French study conducted by Borel et al. in 2006, an RDI < 85% was described for 18.5% of patients treated with R-CHOP who benefited from a phone-based intervention by a certified oncology nurse [32]. The potential impact of the ULP on RDI is particularly interesting, as a better RDI has been associated to improved survival and/or treatment response in several HL [33] and NHL studies [34]. 

From an economic point of view, the ULP was evaluated from the French public health insurance perspective because re-hospitalisation represents a major expenditure. We observed a difference of roughly EUR 81,000 over one year in favour of the ULP group. Including the cost of the ULP implementation, a positive margin of EUR 17,000/year was observed. A more in-depth economic evaluation should be conducted to better evaluate saving and avoided costs achieved through PIs in the ULP, as did de Gregori et al. in cancer patients. When the cost of employing a pharmacist was subtracted from the average yearly cost savings plus cost avoidance per pharmacist, this yielded a net benefit of EUR 223,021 [15]. 

Concerning QoL, even though our results were not statistically significant, there was a trend towards an improvement for many items. This reinforces the benefit of the action carried out by the nurses thanks to the daily follow-up of patients in their homes. 

## 5. Conclusions

To conclude, our study highlights the positive impact of a triple nurse–pharmacist–haematologist collaboration to reduce severe AEs and associated re-hospitalisations through the earlier detection of first symptoms and better management of supportive care in patients receiving highly haematotoxic regimens for HL and NHL. Our results also show trends towards a better ARDI as well as patient satisfaction and improvement in QoL. Further prospective, randomised studies are warranted to demonstrate the clinical and economic impact of this triple collaboration in lymphoma patients receiving highly haematotoxic chemotherapy regimens.

## Figures and Tables

**Table 1:** Characteristics of our UMACOACH Lymphoma population.

Characteristics	All	HL	NHL	NHL Subtypes			
				DLBCL	FL	MCL	Others
Total number of patients	114	19	95	55 (58)	17 (18)	12 (13)	11 (12)
Age (years) Median [Min-Max]	66 [22–92]	34 [21–83]	69 [36–92]	70 [36–92]	62 [45–86]	70 [44–77]	59 [39–82]
Body surface area (m^2^) Median [Min-Max]	1.8 [1.3–2.2]	1.7 [1.36–2.2]	1.8 [1.3–2.2]	1.8 [1.3–2.2]	1.8 [1.5–2]	1.9 [1.6–2]	1.8 [1.5–2]
Gender n (%)							
Male	64 (56)	10 (53)	54 (57)	29 (53)	10 (59)	8 (67)	7 (64)
Female	50 (44)	9 (47)	41 (43)	26 (47)	7 (41)	4 (33)	4 (36)
Ann Arbor stage n (%)							
I–II	25 (22)	12 (63)	13 (14)	10 (18)	1 (6)	0	2 (18)
III–IV	89 (78)	7 (37)	82 (86)	45 (82)	16 (94)	12 (100)	9 (82)
Performance status (ECOG) n (%)							
0–1	90 (80)	16 (84)	74 (79)	41 (75)	14 (88)	11 (92)	8 (73)
2–4	23 (20)	3 (16)	20 (21)	14 (25)	2 (12)	1 (8)	3 (27)
Age adjusted IPI n (%)		-					
0–1	32 (55)	-	32 (55)	30 (55)	-	-	2 (67)
2–3	26 (45)		26 (45)	25 (45)	-	-	1 (33)
Occupational status n (%)							
Active	6 (5)	4 (21)	2 (2)	2 (4)	0	0	0
Inactive/jobless	34 (30)	11 (58)	23 (24)	8 (15)	6 (35)	3 (25)	6 (55)
Retired	74 (65)	4 (21)	70 (74)	45 (82)	11 (65)	9 (75)	5 (45)
Treatment regimens n (%)							
R-CHOP	59 (52)	0	59 (62)	36 (65)	13 (76)	8 (67)	2 (18)
R-miniCHOP	13 (11)	0	13 (14)	10 (18)	3 (18)	0	0
R-ACVBP	8 (7)	0	8 (8)	6 (11)	0	0	2 (18)
BEACOPP	5 (4)	5 (26)	0	0	0	0	0
ABVD	10 (9)	10 (53)	0	0	0	0	0
Others	19 (17)	4 (21)	15 (16)	3 (5)	1 (6)	4 (33)	7 (64)

**Table 2:** Characteristics of cases and controls population.

Characteristics	Cases	Controls
Total number of patients	78	78
Lymphoma type		
HL	12	12
NHL	66	66
DLBCL	42	42
FL	12	12
MCL	10	10
Others	2	2
Age (years) Median [Min-Max]	65 [22–89]	64 [22–96]
Body surface area (m^2^) Median [Min-Max]	1.83 [1.4–2.2]	[1.3–2.2]
Gender n (%)		
Male	44 (56)	40 (51)
Female	34 (44)	38 (49)
Ann Arbor stage n (%)		
I–II	28 (36)	34 (44)
III–IV	50 (64)	44 (56)
Performance status (ECOG) n (%)		
0–1	65 (83)	67 (86)
2–4	13 (17)	11 (14)
Age adjusted IPI n (%)		
0–1	23 (55)	23 (55)
2–3	19 (45)	19 (45)
Occupational status n (%)		
Active	23 (30)	29 (37)
Inactive/jobless	5 (6)	2 (3)
Retired	50 (64)	47 (60)
Treatment regimens n (%)		
R-CHOP	47 (60)	48 (62)
R-miniCHOP	7 (9)	6 (8)
R-ACVBP	6 (8)	6 (8)
BEACOPP	3 (4)	3 (4)
ABVD	7 (9)	7 (9)
Others	8 (10)	8 (10)

**Table 3:** Description of pharmacist interventions (PIs).

		Drug Related Problem (According to FSCP)	n (%)	PI (According to FSCP)	n (%)
**PI category** **n = 300**	PI with prescribers (PIpr) n = 115 (38%)	Contra-indication/non-conformity to guidelines Drug or medical device not received by the patient Dosage problem (under or over dosage) Unjustified drug prescription Drug interaction Adverse drug reaction Improper prescription Drug omission Monitoring Therapeutic redundancy Pharmacodependence	15 (13) 41 (36) 19 (17) 10 (9) 4 (3) 1 (1) 15 (13) 4 (3) 6 (5) 0 0	Addition of a new drug Discontinuation or refusal to deliver Drug switch Choice of administration route Drug monitoring Optimisation of the dispensing/administration mode Dose adjustment	45 (39) 30 (26) 01 (1) 4 (3) 8 (7) 27 (23)
	PI with patients (PIpa) n = 185 (62%)	Improper prescription	185 (100)	Optimisation of the dispensing/administration mode	185 (100)
**Clinical Impact of PI** **n = 300**		Clinical impact of PI (according to CLEO) n (%)			
		Harmful Null Minor Moderate Major Lethal Non determined	0 2 (1) 219 (73) 63 (21) 16 (5) 0 0		

FSCP: French Society for Clinical Pharmacy. Cleo: Clinical Economic and Organizational.

**Table 4:** Treatment adverse events.

	Cases (n = 78)			Controls (n = 78)			*p* Value
Adverse Events	All	Grade 1–2	Grade 3–4	All	Grade 1–2	Grade 3–4	
Anaemia	77 (99)	65 (83)	12 (15)	76 (97)	62 (79)	14 (18)	0.672
Thrombocytopenia	59 (76)	45 (58)	14 (18)	48 (61)	32 (41)	16 (21)	0.288
Neutropenia	77 (99)	2 (3)	75 (96)	78 (100)	1 (1)	77 (99)	0.620
Febrile neutropenia	10 (13)	0	10 (13)	11 (14)	1 (1)	10 (13)	0.524
Infection without neutropenia	27 (35)	22 (28)	5 (6)	25 (32)	13 (17)	12 (15)	0.038
Diarrhea	18 (23)	17 (22)	1 (1)	9 (12)	9 (12)	0	0.667
Constipation	34 (44)	33 (42)	1 (1)	21 (27)	21 (27)	0	0.618
Haemorrhoids	16 (21)	16 (21)	0	7 (9)	7 (9)	0	-
Mucositis	22 (28)	19 (24)	3 (4)	17 (22)	14 (18)	3 (4)	0.535
Neuropathy	31 (40)	31 (40)	0	18 (23)	18 (23)	0	-
Pruritus/eruption	13 (17)	13 (17)	0	8 (10)	8 (10)	0	-
Pulmonary disorders (cough/dyspnea)	18 (23)	18 (23)	0	4 (5)	4 (5)	0	-

**Table 5:** Outcomes in the cases and the controls population.

	Cases (n = 78)	Controls (n = 78)	*p* Value
Number of GCSF injection (total)	1007	949	
Patient receiving GCSF, n (%)	74 (95)	72 (92)	0.746
Patient receiving ASE, n (%)	31 (40)	11 (14)	0.001
Transfusions (total)	140	157	
Transfused patient, n (%)	31 (40)	34 (44)	0.745
Re-hospitalised patients	35 (45)	37 (47)	0.872
Re-hospitalisation (total)	76	88	
Outpatient department, n (%)	54 (71)	53 (60)	0.217
Hospitalisation, n (%)	22 (29)	34 (39)	
Intensive care unit, n (%)	0	1 (1)	
Re-hospitalisation cause			
Febrile neutropenia/Infection, n (%)	17 (22)	29 (33)	0.179
Blood transfusions, n (%)	51 (67)	42 (48)	
ARDI			
<85%, n (%)	6 (8)	13 (18)	0.138
>85%, n (%)	67 (92)	60 (82)	
Delayed treatment (>7 days), n (%)	23 (32)	24 (33)	1

**Table 6:** Satisfaction questionnaire results.

Questions n (%)	I Fully Agree	Moderately Agree	Disagree at All	Not Answered
Have regular phone calls reassured you, put you at ease?	80 (99)	1 (1)	0	0
Did the rhythm of the calls match to the difficulties related to the side effect you experienced?	72 (89)	8 (10)	0	1 (1)
	Much better than expected	As much as expected	A little less than expected	Not answered
Did the listening and the time spent meet to your needs and expectations?	52 (64)	29 (36)	0	0
Were the given answers adapted to your needs?	47 (58)	33 (41)	1 (1)	0
	Very important	Quite important	Little important	Not answered
Was it important for you to be assisted by a health professional in your care pathway (contact, telephone, advice, etc)?	70 (86)	11 (14)	0	0
	Very satisfied	Satisfied	Unsatisfied	Not answered
Are you satisfied with the explanations given by the pharmacist about treatment and their adverse drug effect?	53 (65)	24 (30)	2 (2)	2 (2)
Are you satisfied with the answers given by the pharmacist to your questions?	53 (65)	25 (31)	2 (2)	1 (1)
Are you satisfied with the written information you received (personalised pharmaceutical plan)?	46 (57)	32 (40)	2 (2)	1 (1)
	Yes	No	Not answered	
Has all the support provided by the various people involved (doctor, nurse, pharmacist) helped you to better understand your treatment?	79 (98)	2 (2)	0	
Would you recommend this type of phone follow-up to one of your relative?	79 (98)	2 (2)	0	
In the meantime, between phone appointments, have you encountered any difficulties in contacting the nurse?	7 (9)	74 (91)	0	

**Table 7:** Quality of life results.

		Score		
Items	Visit	Mean	Evolution	*p* Value
Global health status	Before	64.0	3.3	0.298
	After	67.3		
Physical functioning	Before	74.7	3.3	0.514
	After	78.0		
Role functioning	Before	69.5	12.1	0.077
	After	81.6		
Emotional functioning	Before	63.1	4.2	0.241
	After	67.3		
Cognitive functioning	Before	81.6	2.3	0.271
	After	83.9		
Social functioning	Before	71.0	0.6	0.975
	After	71.6		
Fatigue	Before	48.3	−8.8	0.161
	After	39.5		
Nausea and Vomiting	Before	7.5	−1.7	0.590
	After	5.7		
Pain	Before	29.9	−9.2	0.182
	After	20.7		
Dyspnea	Before	20.7	6.9	0.277
	After	27.6		
Insomnia	Before	43.7	−5.7	0.537
	After	37.9		
Appetite Loss	Before	25.3	−8.0	0.300
	After	17.2		
Constipation	Before	29.9	−3.4	0.912
	After	26.4		
Diarrhoea	Before	20.7	−8.0	0.137
	After	12.6		
Financial Difficulties	Before	11.1	−3.7	0.416
	After	7.4		
QLQ-C30 Summary Score	Before	72.2	5.1	0.199
	After	77.3		

## Data Availability

The data presented in this study are available on request from the corresponding author. The data are not publicly available due to University Hospital of Dijon property rules.

## References

[1] Laurent C., Baron M., Amara N., Haioun C., Dandoit M., Maynadié M., Parrens M., Vergier B., Copie-Bergman C., Fabiani B. (2017). Impact of Expert Pathologic Review of Lymphoma Diagnosis: Study of Patients From the French Lymphopath Network. J. Clin. Oncol..

[2] Coiffier B., Lepage E., Brière J., Herbrecht R., Tilly H., Bouabdallah R., Morel P., Van Den Neste E., Salles G., Gaulard P. (2002). CHOP chemotherapy plus rituximab compared with CHOP alone in elderly patients with diffuse large-B-cell lymphoma. N. Engl. J. Med..

[3] Rossi C., Bastie J.N. (2019). Actualités thérapeutiques dans les lymphomes non hodgkiniens et le lymphome de Hodgkin. Rev. Méd. Interne.

[4] Le Gouill S., Thieblemont C., Oberic L., Moreau A., Bouabdallah K., Dartigeas C., Damaj G., Gastinne T., Ribrag V., Feugier P. (2017). Rituximab after Autologous Stem-Cell Transplantation in Mantle-Cell Lymphoma. N. Engl. J. Med..

[5] Fitoussi O., Belhadj K., Mounier N., Parrens M., Tilly H., Salles G., Feugier P., Ferme C., Ysebaert L., Gabarre J. (2011). Survival Impact of Rituximab Combined with ACVBP and Upfront Consolidation Autotransplantation in High-Risk Diffuse Large B-cell Lymphoma for GELA. Haematologica.

[6] Récher C., Coiffier B., Haioun C., Molina T.J., Fermé C., Casasnovas O., Thiéblemont C., Bosly A., Laurent G., Morschhauser F. (2011). Intensified chemotherapy with ACVBP plus rituximab versus standard CHOP plus rituximab for the treatment of diffuse large B-cell lymphoma (LNH03-2B): An open-label randomised phase 3 trial. Lancet.

[7] Italiano A., Jardin F., Peyrade F., Saudes L., Tilly H., Thyss A. (2005). Adapted CHOP plus rituximab in non-Hodgkin’s lymphoma in patients over 80 years old. Haematologica.

[8] Peyrade F., Jardin F., Thieblemont C., Thyss A., Emile J.F., Castaigne S., Coiffier B., Haioun C., Bologna S., Fitoussi O. (2011). Attenuated immunochemotherapy regimen (R-miniCHOP) in elderly patients older than 80 years with diffuse large B-cell lymphoma: A multicentre, single-arm, phase 2 trial. Lancet Oncol..

[9] André M.P.E., Girinsky T., Federico M., Reman O., Fortpied C., Gotti M., Casasnovas O., Brice P., Van der Maazen R., Re A. (2017). Early Positron Emission Tomography Response-Adapted Treatment in Stage I and II Hodgkin Lymphoma: Final Results of the Randomized EORTC/LYSA/FIL H10 Trial. J. Clin. Oncol..

[10] Skoetz N., Will A., Monsef I., Brillant C., Engert A., von Tresckow B. (2017). Comparison of first-line chemotherapy including escalated BEACOPP versus chemotherapy including ABVD for people with early unfavourable or advanced stage Hodgkin lymphoma. Cochrane Database Syst. Rev..

[11] Casasnovas R.O., Bouabdallah R., Brice P., Lazarovici J., Ghesquieres H., Stamatoullas A., Dupuis J., Gac A.C., Gastinne T., Joly B. (2019). PET-adapted treatment for newly diagnosed advanced Hodgkin lymphoma (AHL2011): A randomised, multicentre, non-inferiority, phase 3 study. Lancet Oncol..

[12] Trotman J., Barrington S.F. (2021). The role of PET in first-line treatment of Hodgkin lymphoma. Lancet Haematol..

[13] Pettengell R., Schwenkglenks M., Bosly A. (2008). Association of reduced relative dose intensity and survival in lymphoma patients receiving CHOP-21 chemotherapy. Ann. Hematol..

[14] Yamaguchi H., Hirakawa T., Inokuchi K. (2011). Importance of Relative Dose Intensity in Chemotherapy for Diffuse Large B-cell Lymphoma. J. Clin. Exp. Hematop..

[15] De Grégori J., Pistre P., Boutet M., Porcher L., Devaux M., Pernot C., Chrétien M.L., Rossi C., Manfredi S., Dalac S. (2020). Clinical and economic impact of pharmacist interventions in an ambulatory hematology-oncology department. J. Oncol. Pharm. Pract..

[16] Swerdlow S.H., Campo E., Pileri S.A., Harris N.L., Stein H., Siebert R., Advani R., Ghielmini M., Salles G.A., Zelenetz A.D. (2016). The 2016 revision of the World Health Organization classification of lymphoid neoplasms. Blood.

[17] U.S. Department of Health and Human Resources (2017). Common Terminology Criteria for Adverse Events (CTCAE).

[18] Dalton K., Byrne S. (2017). Role of the Pharmacist in Reducing Healthcare Costs: Current Insights. Integr. Pharm. Res. Pract..

[19] Vo T.H., Bardet J.D., Charpiat B., Leyrissoux C., Gravoulet J., Allenet B., Conort O., Bedouch P. (2018). Validation of a tool for reporting pharmacists’ interventions in everyday community pharmacy. J. Clin. Pharm. Ther..

[20] Stämpfli D., Baumgartner P., Boeni F., Bedouch P., Lampert M.L., Hersberger K.E. (2019). Translation and validation of a tool to assess the impact of clinical pharmacists’ interventions. Int. J. Clin. Pharm..

[21] Vo T.H., Charpiat B., Chanoine S., Juste M., Roubille R., Rose F.X., Conort O., Allenet B., Bedouch P., Working Group “Valorization of Pharmacist Interventions” (2021). Evaluation of the Potential Impact of Pharmacist Interventions: Development and Validation of the CLEO Multidimensional Tool.

[22] Aapro M.S., Bohlius J., Cameron D.A., Dal Lago L., Donnelly J.P., Kearney N., Lyman G.H., Pettengell R., Tjan-Heijnen V.C., Walewski J. (2011). 2010 update of EORTC guidelines for the use of granulocyte-colony stimulating factor to reduce the incidence of chemotherapy-induced febrile neutropenia in adult patients with lymphoproliferative disorders and solid tumours. Eur. J. Cancer.

[23] ASCO Clinical Practice Guideline Update: Recommendations for the Use of White Blood Cell Growth Factors—The ASCO Post. https://www.ascopost.com/issues/november-10-2015/asco-clinical-practice-guideline-update-recommendations-for-the-use-of-white-blood-cell-growth-factors/.

[24] Hryniuk W., Bush H. (1984). The importance of dose intensity in chemotherapy of metastatic breast cancer. J. Clin. Oncol..

[25] Terada Y., Nakamae H., Aimoto R., Kanashima H., Sakamoto E., Aimoto M., Inoue E., Koh H., Nakane T., Takeoka Y. (2009). Impact of relative dose intensity (RDI) in CHOP combined with rituximab (R-CHOP) on survival in diffuse large B-cell lymphoma. J. Exp. Clin. Cancer Res..

[26] Lyman G.H., Dale D.C., Friedberg J., Crawford J., Fisher R.I. (2004). Incidence and predictors of low chemotherapy dose-intensity in aggressive non-Hodgkin’s lymphoma: A nationwide study. J. Clin. Oncol..

[27] Cheson B.D., Pfistner B., Juweid M.E., Gascoyne R.D., Specht L., Horning S.J., Coiffier B., Fisher R.I., Hagenbeek A., Zucca E. (2007). Revised Response Criteria for Malignant Lymphoma. J. Clin. Oncol..

[28] Code de la Santé Publique—Article L710-6. Code de la Santé Publique. https://www.legifrance.gouv.fr/loda/article_lc/LEGIARTI000006694599/1994-10-11.

[29] EORTC Quality of Life Website. https://qol.eortc.org/.

[30] Husson O., de Rooij B.H., Kieffer J., Oerlemans S., Mols F., Aaronson N.K., Van der Graaf W.T.A., Van de Poll-Franse L.K. (2020). The EORTC QLQ-C30 Summary Score as Prognostic Factor for Survival of Patients with Cancer in the “Real-World”: Results from the Population-Based PROFILES Registry. Oncologist.

[31] Osoba D., Rodrigues G., Myles J., Zee B., Pater J. (1998). Interpreting the significance of changes in health-related quality-of-life scores. J. Clin. Oncol..

[32] Borel C., Lamy S., Compaci G., Récher C., Jeanneau P., Nogaro J.C., Bauvin E., Despas F., Delpierre C., Laurent G. (2015). A longitudinal study of non-medical determinants of adherence to R-CHOP therapy for diffuse large B-cell lymphoma: Implication for survival. BMC Cancer.

[33] Russo F., Svanera G., Cioppa P.D., Corazzelli G., Frigeri F., Capobianco G., LaStoria S., Pinto A. (2005). The Impact of Relative Dose Intensity on Response and Survival in a Series of 180 Newly Diagnosed Patients with Hodgkin’s Lymphoma. Blood.

[34] Bosly A., Bron D., Van Hoof A., De Bock R., Berneman Z., Ferrant A., Kaufman L., Dauwe M., Verhoef G. (2008). Achievement of optimal average relative dose intensity and correlation with survival in diffuse large B-cell lymphoma patients treated with CHOP. Ann. Hematol..

